# Unexpected rare uterine carcinosarcoma with neuroendocrine differentiation: Reflections on clinical diagnosis and treatment of a case report

**DOI:** 10.1097/MD.0000000000038800

**Published:** 2024-07-12

**Authors:** Qichong Shi, Longmei Wang, Juan Yao

**Affiliations:** a Department of General Surgery, The Fifth People’s Hospital of Huaian, Yangzhou University Huaian, Jiangsu, China; b Department of Pathology, The Fifth People’s Hospital of Huaian, Yangzhou University Huaian, Jiangsu, China.

**Keywords:** carcinosarcoma, endometrial, heterologous, small cell neuroendocrine carcinoma

## Abstract

**Rationale:**

Uterine carcinosarcoma (UCS) is a rare and highly invasive malignant tumor.It exhibits an ectopic growth pattern of the uterus,and its histological features are biphasic differentiation of malignant epithelial components (cancer) and malignant mesenchymal components (sarcoma). The pathological pattern of high-component neuroendocrine differentiation is extremely rare. Due to the inherent heterogeneity of tumors, it increases the difficulty of accurate identification and diagnosis. The author introduces a rare case of primary endometrial carcinosarcoma (heterologous) with small cell neuroendocrine carcinoma (SCNEC) components. There is limited literature on this rare pathological differentiation pattern and a lack of guidelines for the best treatment methods, which prompts reflection on the diagnosis, optimal treatment strategies, and how preoperative diagnosis can affect patient prognosis for endometrial carcinosarcoma with neuroendocrine differentiation.

**Patient concerns:**

The patient is an elderly woman who presents with abnormal vaginal bleeding after menopause. Transvaginal ultrasound examination shows that the uterus is slightly enlarged, and there is a lack of homogeneous echogenicity in the uterine cavity. Subsequently, a hysteroscopic curettage was performed, and a space-occupying lesion was observed on the anterior wall of the uterine cavity.

**Diagnoses:**

Preoperative endometrial biopsy revealed SCNEC of the endometrium. The patient underwent radical hysterectomy, and the postoperative pathological results showed that UCS (heterologous) was accompanied by SCNEC components (about 80%).

**Intervention:**

The patient received radical hysterectomy, followed by adjuvant chemotherapy.

**Outcome:**

After 7 months of follow-up, no tumor recurrence or metastasis was found at the time of writing this article.

**Lessons:**

The histological type of UCS (heterologous) with cell neuroendocrine carcinoma components is rare and highly invasive, with a high misdiagnosis rate in preoperative biopsy. There are currently no effective treatment guidelines for this type of case. The unusual appearance of SCNEC components in this case poses a challenge for both pathologists and surgeon. The rare differentiation pattern of this case exposes the complexity of its management and the necessity of prospective trials to determine the optimal treatment plan.

## 1. Introduction

Uterine carcinosarcoma (UCS) is a rare subtype of cancer, accounting for <5% of primary malignant tumors of the uterus.^[1]^ The pathological pattern of high-component neuroendocrine differentiation is extremely rare. Research has shown that these different histological sources are classified into homologous (leiomyosarcoma, fibrosarcoma, endometrial stromal sarcoma) and heterologous (rhabdomyosarcoma, bone and chondrosarcoma).^[2]^ Due to the inherent heterogeneity of tumors, it is difficult for biopsy materials to fully capture multiple components, which increases the difficulty of accurate identification and diagnosis. Diagnosis is usually clear after surgery and prognosis is poor. However, there is very little literature on this type of UCS with special neuroendocrine differentiation, and there is a lack of guidelines for the best treatment methods. Given its heterogeneity, prognosis largely depends on the most invasive components of tumor staging and differentiation types.^[3]^

## 2. Case presentation

**Figure 1.**
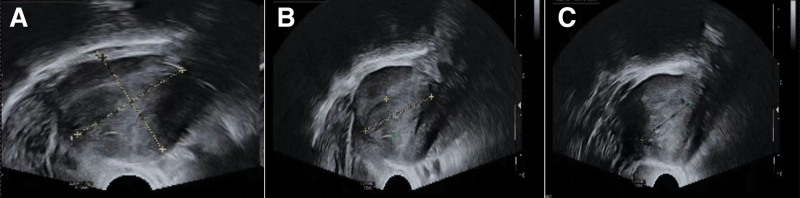
(A–C) Transvaginal ultrasound (TVS) examination shows that the uterus is slightly enlarged, and there is a lack of homogeneous echogenicity in the uterine cavity.

**Figure 2.**
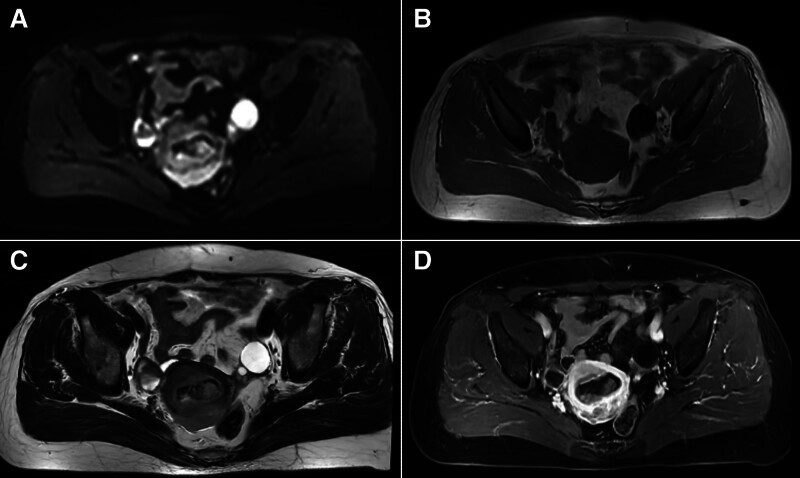
(A–D) Abdominal magnetic resonance imaging (MRI) shows that the uterus is slightly enlarged, with irregular morphology, uneven signal in the uterine cavity, and multiple patchy soft tissue nodules. DWI shows an uneven high signal, T1WI shows isolow signal, T2WI shows a mixed high and low signal, with unclear edges. The enhancement scan is uneven, with a degree of enhancement lower than that of the normal uterine muscle layer, and there is a patchy area without enhancement. DWI = diffusion weighted imaging, T1WI = T1-weighted imaging, T2WI = T2-weighted imaging.

**Figure 3.**
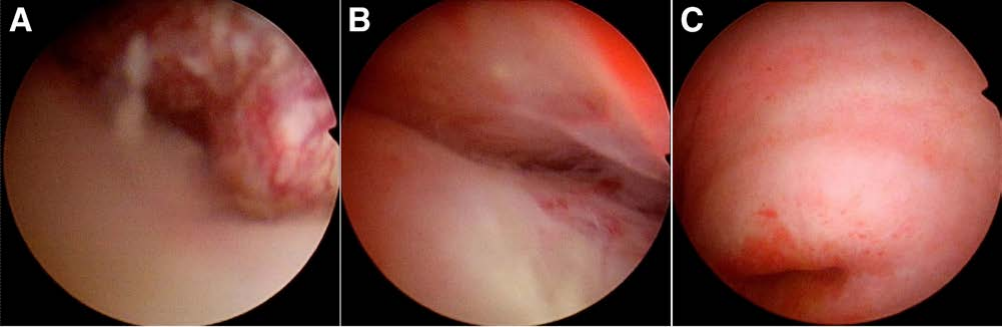
(A–C) Hysteroscopy revealed a space-occupying a lesion in the anterior wall of the uterine cavity, with irregular surface blood vessels.

A 60-year-old female patient had abnormal uterine bleeding after menopause, and laboratory tests showed an increase in serum alpha-fetoprotein index (101.88 ng/mL, reference level 0–7 ng/mL). Transvaginal ultrasound examination shows that the uterus is slightly enlarged, and there is a lack of homogeneous echogenicity in the uterine cavity (Fig. 1). Abdominal magnetic resonance imaging shows that the uterus is slightly enlarged, with irregular morphology, uneven signal in the uterine cavity, and multiple patchy soft tissue nodules. The uterine serous layer is still intact, and no space-occupying lesions are found in the cervix, vagina, and bilateral appendages. There are no obvious enlarged lymph nodes in the pelvic cavity (Fig. 2). Computer tomography (CT) did not show any occupying lesions or distant metastases in other areas. Subsequently, a hysteroscopic curettage was performed, and a space-occupying lesion was observed on the anterior wall of the uterine cavity, with surface blood vessels dilated and irregularly oriented (Fig. 3). The biopsy pathology shows small cell neuroendocrine carcinoma (SCNEC) of the endometrium.

The patient underwent laparoscopic transabdominal hysterectomy, pelvic and abdominal systematic lymphadenectomy, and bilateral ovarian and salpingectomy. During the operation, it was recorded that the uterus was slightly enlarged, the serous layer of the posterior wall of the uterus was slightly raised, and the lesion did not break through the serous layer. No obvious masses were found in the bilateral appendages.

**Figure 4.**
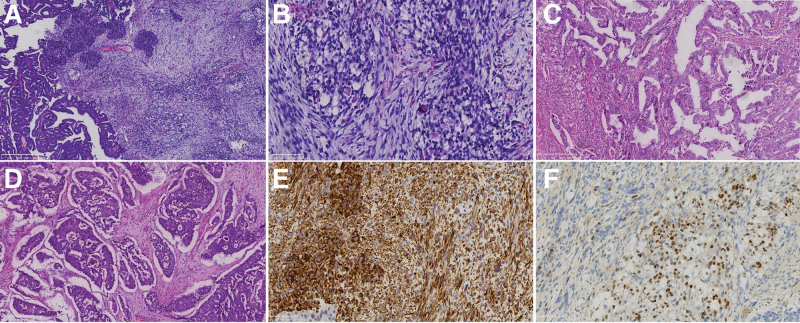
Pathological analysis of tumor tissue. (A) The tumor tissue consists of a mixture of 2 components: cancer and sarcoma (H&E, original magnification × 40). (B) In the area of mesenchymal components, tumor cells are densely arranged in a spindle shaped. Circular and oval rhabdomyoblast are scattered among them. Nucleolus and myofilament-like structures can be seen (H&E, original magnification × 200). (C) In the epithelial component area, tumor cells exhibit glandular tubular or papillary structures, with complex glandular hyperplasia and crowded arrangement (H&E, original magnification × 100). (D)The tumor is composed of round to oval-shaped cells, growing in a solid nest shape with sparse chromatin and deeply stained nuclei (H&E, original magnification × 100). (E–F) Positive immunohistochemical staining for Desmin and MyoD1 (IHC, original magnification × 200). H&E = hematoxylin-eosin, IHC = Immunohistochemistry.

**Figure 5.**
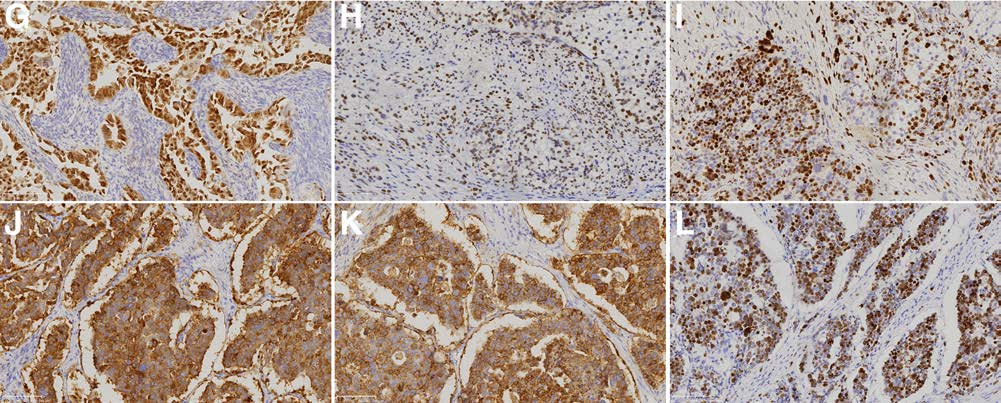
IHC analysis of tumor tissue. (G–I) Tumor cells with glandular tubular arrangement express Pax-8, P53, and Ki-67 (proliferation index of approximately 60%) (IHC, original magnification × 200). (J–L) The tumor cells in a solid nest-like arrangement express Syn, CD56, and Ki-67 (proliferation index of about 60%) (IHC, original magnification × 200). IHC = immunohistochemistry.

The pathological examination results of the surgical specimen show that in the interstitial area, tumor cells are arranged in a spindle shape, with easily visible mitotic figures and mucinous stroma. Spider-like rhabdomyocytes are scattered among them, and larger atypical tumor giant cells can be seen (Fig. 4). Immunohistochemical staining showed positive staining for Desmin, Vimentin, MyoD1, Myogenin, Myolobin, P53, and Ki-67, while SMA was negative. In the epithelial component area, some tumor cells exhibit glandular tubular, papillary, and sieve-like structures, with complex glandular hyperplasia and crowded arrangement. Immunohistochemical staining shows positive expression of Pax-8 and P53. In addition, the majority of the tumor is composed of round to oval cells, and immunohistochemical staining shows positive staining for Syn, CD56, and Ki-67 (Fig. 5). Due to the extremely rare nature of the case, pathological sections were sent for second opinion, and combined with cell morphology and immunohistochemical results, the diagnosis of primary UCS (heterologous) with SCNEC components (approximately 80%) was ultimately confirmed. The tumor tissue invaded the entire layer of the uterine muscle wall and vascular invasion was observed. Cancer tissue metastasis was observed in pelvic and abdominal lymph nodes, no invasion of cancer tissue was found in the cervix, bilateral appendages, and greater omentum. Hence, the tumor was classified as a pT3aN2aM0.

There are currently no effective treatment guidelines for this type of case, which poses a challenge to clinical treatment. According to the recommendations of the multidisciplinary oncology committee, the patient is currently undergoing postoperative adjuvant chemotherapy (EP).

## 3. Discussion

UCS is a heterogeneous and highly invasive malignant biphasic tumor, with SCNEC giving it higher invasiveness and a poorer prognosis trend.^[4]^ In recent years, endometrial carcinosarcoma has attracted much attention due to its genetic heterogeneity and molecular variation, leading to differences in clinical treatment responsiveness.^[5]^ Due to the characteristics of tumors that display both epithelial and mesenchymal components under a microscope, most endometrial cancers have similar imaging features, which increases the difficulty of accurate identification and diagnosis. Especially in small endometrial biopsy specimens, the lesion may be misdiagnosed due to insufficient sampling. In limited biopsy specimens where mesenchymal components are not present, It is highly likely to be misdiagnosed as neuroendocrine carcinoma or poorly differentiated endometrioid carcinoma. The unusual appearance of SCNEC components in this case poses a challenge for both pathologists and surgeon.

Some cases that may cause diagnostic differences also need to consider Müllerian adenosarcoma of the uterus and high-grade endometrial stromal sarcoma (HGESS). The characteristic of Müllerian adenosarcoma is the presence of benign epithelial glandular components, with the sarcoma surrounding the gland and exhibiting polypoid overgrowth.^[6]^ This typical histological feature enables it to be distinguished from UCS. HGESS is a type of stromal tumor with genetic diversity, composed of well-differentiated circular or spindle-shaped cells, distributed in a nodular growth pattern around spiral blood vessels, without cancer components.^[7]^ Keratin immunostaining is helpful in distinguishing between the 2. The lack of specific biomarkers for UCS limits rapid clinical diagnosis and personalized treatment strategies.

The author believes that preoperative misdiagnosis as endometrial SCNEC does not pose a risk of inadequate treatment during the surgical process. In fact, UCS has previously considered a Müllerian duct tumor and was classified as endometrial cancer in the 2003 World Health Organization (WHO) classification of female reproductive tract tumors.^[8]^ Endometrial neuroendocrine carcinoma is highly invasive, and compared to other types of endometrial cancer, it has a poorer prognosis (total survival period of 12–22 months) and a higher risk of death (95% CI: 1.88–2.88%).^[9]^ The standard treatment methods include radical hysterectomy, bilateral salpingo-oophorectomy, pelvic lymph node dissection, and postoperative adjuvant radiotherapy and chemotherapy.^[10]^ This type of tumor meets the anatomical criteria for surgical resection, and radical surgery with extensive resection remains an active treatment for UCS with small cell neuroendocrine differentiation.^[1]^

Due to the rare incidence rate of such cases, the lack of available data on their prognostic impact, and the difficulty in obtaining accurate histopathology before surgery, there is only one relevant phase 3 trial in this case.^[11]^ But so far, no prospective studies have shown that preoperative adjuvant radiotherapy or chemotherapy is beneficial for the overall survival of patients with UCS.^[12]^

Still, accurate preoperative diagnosis of carcinosarcoma may be valuable in assessing metastatic environments, as chemotherapy regimens should target metastatic differentiation components.

Given the lack of high-quality evidence and sample data to support prospective studies on large-scale chemotherapy regimens, the optimal chemotherapy strategy remains controversial. The rarity of such tumors and the trend toward accurate diagnosis only after resection exacerbate this situation. In addition, the presence of multiple special differentiation patterns increases complexity. The effectiveness of postoperative adjuvant therapy for heterologous UCS may be related to the epithelial and sarcoma factors of the primary tumor.^[13]^ Given the poor prognosis associated with carcinosarcoma, existing studies recommend multimodal comprehensive treatment, including postoperative chemotherapy and radiation therapy, for resectable individuals.^[14]^ The data guiding chemotherapy mainly comes from retrospective studies and experiences of epithelial cancer.^[15]^ Romeo et al emphasized that postoperative adjuvant chemotherapy has a positive impact on the survival rate of all stages of UCS, as well as the importance of combining platinum-based drugs to treat recurrence.^[12]^ The chemotherapy regimen based on cisplatin is most commonly used for patients with UCS at different stages.^[16]^ Research has shown that compared to combination therapy based on ifosfamide, the carboplatin-paclitaxel chemotherapy regimen is recommended due to its controllable safety and comparable progression-free survival.^[17]^ There is limited data on follow-up chemotherapy for advanced stages. It is worth noting that a retrospective series of studies have shown the anti-tumor activity of postoperative chemotherapy (EP) on endometrial SCNEC.^[4]^ Adjuvant chemotherapy (EP) is recommended as a treatment algorithm for endometrial SCNEC.^[18]^ In fact, there are differences in the priority chemotherapy regimens used in existing studies, reflecting the difficulty in predicting the natural course of such diseases, as it relies on multiple complex histological types of differentiation, and there are no prospective randomized controlled trials indicating the optimal sequence of chemotherapy regimens. Considering that the proportion of SCNEC in the patient uterine tumor is as high as 80%, accompanied by heterologous differentiation of rhabdomyosarcoma, the author chose to administer chemotherapy (EP) after surgery. Currently, the patient shows no signs of recurrence. Combining multimodal treatment methods such as surgery and chemotherapy may improve survival outcomes.

## 4. Conclusion

The histological type of UCS (heterologous) with SCNEC components is rare and highly invasive, with a high misdiagnosis rate in preoperative biopsy. There is a lack of consensus on evidence-based guidelines for effective treatment. Early diagnosis, radical surgery combined with multimodal adjuvant therapy, and regular follow-up seem to be still a positive approach. The rare differentiation pattern of this case exposes the complexity of its management and the necessity of prospective trials to determine the optimal treatment plan. With the deepening of research on potential biological and molecular variations, molecular targeting strategies and immunotherapy may become attractive clinical treatment options.

## Author contributions

**Conceptualization:** Qichong Shi, Longmei Wang, Juan Yao.

**Data curation:** Qichong Shi, Longmei Wang, Juan Yao.

**Formal analysis:** Qichong Shi, Longmei Wang.

**Investigation:** Qichong Shi, Longmei Wang, Juan Yao.

**Project administration:** Qichong Shi.

**Resources:** Qichong Shi, Longmei Wang.

**Writing – original draft:** Qichong Shi.

**Writing – review & editing:** Longmei Wang, Juan Yao.
